# Simulation Effect and Mechanism of High-Polymeric Persimmon Tannin on Simulating Alternate-Day Fasting on Regulating Lipid Metabolism in Obese Mice

**DOI:** 10.3390/nu18101608

**Published:** 2026-05-18

**Authors:** Yajie Zhang, Yunfei Huang, Yawei Xu, Chunmei Li

**Affiliations:** College of Food Science and Technology, Huazhong Agricultural University, Wuhan 430070, China

**Keywords:** persimmon tannin, obesity, alternate day fasting

## Abstract

**Background/Objectives:** Obesity represents a significant global health challenge. Although alternate-day fasting (ADF) has been shown to effectively improve metabolic parameters, long-term adherence to this regimen remains limited. This study aimed to investigate whether highly polymerized persimmon tannin (DP31) could serve as a practical alternative to ADF for the prevention of high-fat diet (HFD)-induced obesity in mice. **Methods:** Male C57BL/6J mice (*n* = 10 per group) were subjected to an HFD for 11 weeks, during which they concurrently received either DP31 or ADF. Body weight, fat mass, serum lipid levels, glucose tolerance, fasting glucose, and insulin levels were assessed. Additionally, hepatic transcriptomics, Western blotting, 16S rRNA sequencing, and short-chain fatty acids (SCFAs) analysis were conducted. **Results:** DP31 demonstrated comparable efficacy to ADF in reducing body weight gain and improving lipid profiles, while exhibiting superior effects on glucose tolerance and fasting glucose levels (*p* < 0.05). Both interventions effectively reversed HFD-induced hepatic gene dysregulation, leading to the upregulation of genes involved in processes related to steroid metabolism. In addition, both treatments activated the hepatic AMPK-mTORC1-Lpin1 axis, suppressed lipogenesis, upregulated PGC1α, and increased β-hydroxybutyrate levels, indicating enhanced fatty acid oxidation (*p* < 0.05). Notably, DP31 outperformed ADF in enriching beneficial gut genera, such as *Akkermansia*, and boosting SCFAs production, which may elucidate its superior glycemic control. Overall, DP31 exhibits comparable effects to ADF in preventing obesity-related metabolic disorders, while demonstrating superior effects on glucose homeostasis.

## 1. Introduction

Obesity, defined as a body mass index (BMI) greater than 30 kg/m^2^, is a major risk factor for type 2 diabetes, cardiovascular diseases, cancer, and numerous other metabolic complications [1]. Current therapeutic strategies, including pharmacological treatment and bariatric surgery, present notable limitations. Pharmacological interventions may raise safety concerns due to potential long-term adverse effects [2], whereas surgical approaches are invasive and expensive. Consequently, the development of safe, effective, and accessible alternatives to conventional weight-loss interventions has become a major priority in public health research.

Intermittent fasting (IF) has emerged as an effective non-pharmacological strategy for the prevention and management of chronic metabolic disorders [3]. Among the various IF regimens, ADF, characterized by alternating periods of ad libitum feeding and energy restriction, has demonstrated substantial efficacy in promoting weight loss and reducing visceral adiposity within 4–12 weeks [4]. Previous studies have shown that 8 weeks of ADF treatment significantly reduced inflammation and oxidative stress in overweight adults with asthma [5]. Furthermore, ADF alleviated insulin resistance and hepatic lipid accumulation in db/db mice [6]. However, ADF has been associated with mild adverse effects, including gastrointestinal discomfort, sleep disturbances, and dizziness [7]. In addition, animal studies have suggested potential risks such as cardiac dysfunction under prolonged ADF intervention [8], and long-term adherence to this regimen remains challenging [9]. For many individuals, a 24 h complete fast poses substantial physical and psychological challenges, including intense hunger, irritability, fatigue, and difficulty concentrating, which frequently lead to withdrawal from the dietary regime. Therefore, alternative strategies that can mimic the metabolic benefits of ADF without requiring sustained caloric restriction are of considerable interest.

Accumulating evidence indicates that polyphenolic compounds play an important role in the prevention and management of obesity-related metabolic disorders [10]. Structurally, polyphenols are characterized by one or more hydroxyl groups attached to aromatic rings and exhibit substantial chemical diversity, ranging from simple phenolic acids to highly polymerized compounds such as tannins [11]. To date, most polyphenols proposed as caloric restriction mimetics are low-molecular-weight compounds, including curcumin, catechins, and resveratrol [12]. In recent years, multiple clinical trials have evaluated the caloric restriction-mimetic effects of curcumin in humans. A meta-analysis of eight randomized controlled trials showed that long-term curcumin intervention may reduce total and visceral fat but is insufficient to significantly lower body weight or body mass index [13]. Resveratrol is one of the most well-known caloric restriction-mimetic polyphenols and improves health through multiple mechanisms [14,15]. Tauriainen et al. [16] compared the effects of caloric restriction and resveratrol supplementation on diet-induced obesity and hepatic steatosis, and found that resveratrol did not affect energy intake, weight gain, or body fat percentage, providing only modest protection against hepatic steatosis and hepatocyte ballooning. In contrast, caloric restriction completely prevented obesity and hepatic steatosis, suggesting that higher doses of resveratrol or longer treatment durations may be required. Therefore, it is necessary to identify more suitable natural products that achieve caloric restriction-like effects on lipid metabolism.

Persimmon tannin is a highly polymerized polyphenol derived from persimmon fruit that has been reported to inhibit intestinal glucose utilization and lipid absorption [17,18]. Through modulation of signaling pathways involved in lipid synthesis and oxidation, persimmon tannin has been shown to reduce hepatic lipid accumulation induced by a high-cholesterol diet and to alleviate hepatic steatosis [19]. Notably, as a highly polymerized polyphenol, persimmon tannin may possess distinct bioactivity. Previous studies have shown that highly polymerized proanthocyanidins are more effective than their low-polymerized counterparts in improving intestinal barrier function and alleviating epithelial inflammation [20]. Similarly, it has been reported that proanthocyanidins with a degree of polymerization greater than 10 exert stronger effects on digestive enzyme activity and nutrient absorption than those with a degree of polymerization of 2–4 [21]. These findings support the notion that highly polymerized polyphenols may offer superior metabolic benefits. Therefore, on specific parameters, persimmon tannin has the potential to serve as a substitute for ADF in regulating lipid metabolism.

The research question of this study was whether highly polymerized persimmon tannin could substitute for ADF in regulating lipid metabolism in obese mice. Therefore, we hypothesized that highly polymerized persimmon tannin could substitute for ADF in specific parameters by mimicking its metabolic effects, namely, activation of the hepatic AMPK-mTORC1-Lpin1 signaling axis and modulation of the gut microbiota. The specific objectives were to investigate the effects of highly polymerized persimmon tannin on lipid metabolism and glucose homeostasis in HFD-induced obese mice and to elucidate the underlying molecular mechanisms. To test this hypothesis, we employed transcriptomic analysis, 16S rRNA gene sequencing, and biochemical assays. This work aims to provide a theoretical and experimental basis for the use of highly polymerized persimmon tannin as a practical nutritional strategy for lipid-lowering and weight management.

## 2. Materials and Methods

### 2.1. Materials and Reagents

The persimmon fruit was acquired from the Yangling Persimmon Germplasm Resource Garden in Shaanxi (Yangling, China). Anhydrous ether, methanol and concentrated sulfuric acid were purchased from Sinopharm Chemical Reagent Co., Ltd. (Shanghai, China); AB-8 macroporous adsorption resin was purchased from Nankai University Chemical Plant (Tianjin, China); 60 kcal% high-fat feed (D12492) and D12450J were purchased from Shenzhen Ruidi Biotechnology Co., Ltd. (Shenzhen, China); AMPKα, p-AMPKα, SCD1 and FAS were purchased from Cell Signaling Technology (Danvers, MA, USA); mTOR, p-mTOR, Raptor, p-Raptor, PGC1α, GAPDH, LaminB1 and Lpin1 were purchased from Wuhan ABclonal Biotechnology Co., Ltd. (Wuhan, China); SREBP1 was purchased from Chengdu Zhengneng Biotechnology Co., Ltd. (Chengdu, China); insulin ELISA and β-hydroxybutyric acid kits were purchased from Wuhan Elabscience Biotechnology Co., Ltd. (Wuhan, China); cell nuclei Protein and cytoplasmic protein preparation kits were purchased from Beijing Applygen Gene Technology Co., Ltd. (Beijing, China); TG, T-CHO, HDL-C, LDL-C, glucose and FFA kits were purchased from Nanjing Jiancheng Bioengineering Institute (Nanjing, China); 2-diethylbutyric acid, acetic acid, propionic acid, and butyric acid were purchased from Shanghai Aladdin Biochemical Technology Co., Ltd. (Shanghai, China).

### 2.2. Preparation of Persimmon Tannin

High-polymerized persimmon tannin was extracted from freeze-dried persimmon powder using ultrasonic-assisted methanol extraction, followed by refluxing at 80 °C for three intervals of 30 min each, with a solid-to-liquid ratio of 1:8 (*m*/*v*). The resulting pomace was subsequently extracted with a 1% HCl-methanol solution at the same ratio and at 60 °C for three 30 min intervals. The combined filtrate was concentrated and adsorbed onto AB-8 macroporous resin for 40 min, followed by washing with distilled water until sugar-free, and rinsing with 10% ethanol until colorless to eliminate small phenolic acids. The target compounds were eluted with 95% ethanol, and the eluate was vacuum-evaporated at 35 °C and freeze-dried, resulting in high-polymerized persimmon tannin with a polymerization degree of 31.

### 2.3. Animal Model Establishment and Sample Collection

Six-week-old male SPF grade C57BL/6J mice (*n* = 40) were purchased from Huazhong Agricultural University Experimental Animal Center (Wuhan, China). All mice were healthy, immunocompetent, wild-type (no genetic modification), and had no history of prior experimental procedures. All mice were handled under standard laboratory conditions (temperature 22 ± 1 °C, humidity 55 ± 10% and 12 h light-dark cycle). After one week of adaptive feeding, mice were randomly divided (using a simple randomization procedure) into the following four groups (10 mice in each group, 5 per cage. The sample size of 10 mice per group was selected based on previous publications in the field of obesity and lipid metabolism, in which 8–12 mice per group are commonly used to detect statistically significant differences. This sample size also accounts for potential individual variability.): Group C (normal control group): fed D12450J normal diet + daily gavage of distilled water; Group M (high-fat model group): fed D12492 high-fat diet + daily gavage of distilled water; Group D31 (DP31 intervention group): fed D12492 high-fat diet + daily gavage of 100 mg/kg DP31; Group ADF (alternate-day fasting intervention group): fed D12492 high-fat diet with an alternate-day fasting regimen (24 h ad libitum feeding alternating with 24 h fasting), daily gavage of distilled water.

D12450J feed formula: 20.0 kcal% protein; 70.0 kcal% carbohydrate; 10.0 kcal% fat. D12492 feed formula: 20.0 kcal% protein; 20.1 kcal% carbohydrate; 59.9 kcal% fat. Gavage procedures were performed by the same investigator each morning; body weight and blood glucose measurements were conducted in the morning to control for circadian rhythm; cages of each group were randomly arranged on the racks. No animals died or became moribund during this period, and all completed the experiment. After the 11-week intervention, the mice were fasted for 12 h, after which blood, tissues, and colonic contents were collected. Blood was collected from the retro-orbital sinus and allowed to stand at room temperature for 2 h. Serum was separated by centrifugation at 3000 rpm for 10 min at 4 °C, then aliquoted and transferred to a −80 °C freezer until analysis. The tissues were either fixed in paraformaldehyde or snap-frozen in liquid nitrogen and subsequently stored at −80 °C for further analysis. Similarly, the colonic contents were snap-frozen and preserved at −80 °C for subsequent gut microbiota profiling. Mice experiments were approved by the Experimental Animal Care and Use Committee of Huazhong Agricultural University (Approval Code: HZAUMO-2021-0132), and were conducted following the Guidelines for Care and Use of Laboratory Animals. Mice were monitored daily for signs of distress, pain, or illness, including changes in body weight, food intake, activity level, coat condition, and posture. No adverse events or unexpected deaths occurred during the study. No specific humane endpoints were triggered, as all animals remained healthy throughout the experiment. All investigators were aware of group allocation during the conduct of the experiment, outcome assessment, and data analysis.

### 2.4. Oral Glucose Tolerance Test

At week 8, mice were fasted for 12 h before a glucose solution (2 g/kg) was administered by oral gavage. Tail blood was used to measure glucose concentration with glucose meter at 0, 15, 30, 60, 90, and 120 min. The corresponding values were recorded, and the area under the blood glucose curve (AUC) was calculated.

### 2.5. Detection of Lipid Metabolism Related Indicators

Serum levels of triglycerides (TG), total cholesterol (T-CHO), high-density lipoprotein cholesterol (HDL-C), low-density lipoprotein cholesterol (LDL-C), and glucose were measured using commercial kits in accordance with the manufacturer’s instructions. Insulin (Cat. No. E-EL-M3119) and β-hydroxybutyrate (Cat. No. E-BC-K785-M) were analyzed using a mouse insulin ELISA kit and a β-hydroxybutyrate colorimetric kit (Wuhan Elabscience Biotechnology Co., Ltd., Wuhan, China), respectively, following the recommended protocols.

### 2.6. InAlyzer Assay

The body composition of the mice was assessed using dual-energy X-ray absorptiometry (InAlyzer, Medikors Inc., Seongnam, Republic of Korea). Following anesthesia, the mice were positioned in the InAlyzer scanning area for whole-body scanning. The InAlyzer software (version 1.0.0.0) was utilized to capture the images.

### 2.7. Liver and Colon Sections

HE staining involved dehydrating the liver tissue through a gradient dehydration process, embedding it in paraffin, cutting it into 4 μm thick slices, staining with hematoxylin and eosin, and sealing with a neutral resin film. The liver tissue slices were then observed under an optical microscope using a Nikon inverted microscope (Nikon Corporation, Tokyo, Japan).

### 2.8. Hepatic Transcriptome Analysis

Total RNA was extracted from tissue samples using TRIzol^®^ Reagent (Thermo Fisher Scientific, Waltham, MA, USA) according to the manufacturer’s instructions. Messenger RNA (mRNA) was enriched using magnetic beads, fragmented into approximately 300 base pairs, and reverse-transcribed into double-stranded complementary DNA (cDNA). Following adapter ligation and size selection, the libraries were amplified via PCR, quantified using the QuantiFluor^®^ dsDNA System (Promega Corporation, Madison, WI, USA), and sequenced on an Illumina NovaSeq 6000 platform (Illumina, Inc., San Diego, CA, USA). The sequencing data were aligned with the reference genome for transcript quantification and subsequently subjected to Gene Ontology (GO) and Kyoto Encyclopedia of Genes and Genomes (KEGG) pathway analysis.

To identify DEGs between two different samples, the expression level of each transcript was calculated according to the transcripts per million reads (TPM) method. RSEM was used to quantify gene abundances. Differential expression analysis was performed using DESeq2. DEGs with |log2*FC*| ≥ 1 and *FDR* < 0.05 (DESeq2) or *FDR* < 0.001 (DEGseq) were considered significantly differentially expressed. R software (Version 1.3.3) was used for DESeq2 analysis. In addition, functional enrichment analysis, including GO and KEGG, was performed to identify which DEGs were significantly enriched in GO terms and metabolic pathways at a Bonferroni-corrected *p*-value < 0.05 compared with the whole-transcriptome background. GO functional enrichment and KEGG pathway analysis were carried out by Goatools (Version 0.6.5) and Python scipy software (Version 3.14.5), respectively.

### 2.9. Liver Tissue Protein Extraction and Target Protein Content Determination

Liver tissues from mice were homogenized in ice-cold RIPA buffer (Servicebio, Wuhan, China), which contained phosphatase and protease inhibitors. Following centrifugation at 12,000× *g* for 15 min at 4 °C, the supernatant was collected for Western blot analysis. Nuclear proteins were extracted using a commercial nuclear and cytoplasmic protein preparation kit. Proteins were separated by SDS-PAGE, transferred to PVDF membranes, and blocked for 1 h. The membranes were incubated overnight at 4 °C with primary antibodies against AMPKα, p-AMPKα, GAPDH, mTOR, p-mTOR, PGC1α, Lamin B1, Raptor, p-Raptor, Lpin1, FAS, SCD1, and SREBP1. After washing with TBST, the membranes were incubated with a secondary antibody for 1 h. Protein signals were detected using enhanced chemiluminescence and imaged with an Odyssey Fc Imaging System (LI-COR Biosciences, Lincoln, NE, USA). Quantification was performed using ImageJ software (version 1.54f).

### 2.10. Quantitative Real-Time PCR (RT-qPCR)

Total RNA was extracted using Servicebio (Servicebio, Wuhan, China), and cDNA was synthesized with the SweScript All-in-One SuperMix (Servicebio, Wuhan, China), which includes gDNA removal, under the following conditions: 25 °C for 5 min, 42 °C for 30 min, and 85 °C for 5 s. qPCR was conducted in 15 µL reactions containing SYBR Green Master Mix, gene-specific primers, and the synthesized cDNA. The primers now included are: *Lpin1*, Forward: 5′-CCCTCGATTTCAACGTACCC-3′, Reverse: 5′-GCAGCCTGTGGCAATTCA-3′); *Lpin1α*, Forward: 5′-GGTCCCCCAGCCCCAGTCCTT-3′, Reverse: 5′-GCAGCCTGTGGCAATTCA-3′); and *β-actin*, Forward: 5′-TGCTGTCCCTGTATGCCTCTG-3′, Reverse: 5′-TTGATGTCACGCACGATTTCC-3′ [22]. All samples were analyzed in triplicate under standard cycling conditions: 95 °C for 30 s, followed by 40 cycles of 95 °C for 15 s and 60 ° C for 30 s, accompanied by a melting curve analysis.

### 2.11. Intestinal Flora Diversity Analysis

Total DNA was extracted from fecal samples using the EZNA^®^ Soil DNA Kit (Omega Bio-tek, Norcross, GA, USA). The V3-V4 variable region of the 16S rRNA gene was PCR amplified with the upstream primers 338F (5′-ACTCCTACGGGGAGGCAGCAG-3′) and 806R (5′-GGACTACHVGGGTWTCTAAT-3′). Sequencing was performed using the Illumina MiSeq PE300 platform (Illumina, San Diego, CA, USA). Data analysis was conducted on the Majorbio cloud platform.

### 2.12. Determination of Fecal Short-Chain Fatty Acid Content

Mouse feces were treated with an internal standard (2-ethylbutyric acid) and ultrapure water. The sample was vortexed and then centrifuged. The supernatant was filtered, and 50% concentrated sulfuric acid was added. The mixture was thoroughly mixed and subsequently extracted with ether, followed by another vortexing and centrifugation. The supernatant was collected for analysis. The quantification was performed using gas chromatography with a DB-FFAP chromatographic column (30.0 m × 250 μm × 0.25 μm) from Agilent Technologies Inc., Santa Clara, CA, USA. The injection volume was 1 μL with a split ratio of 10:1. The initial temperature was set at 105 °C and increased to 230 °C at a rate of 10 °C/min, where it was held for 2 min. The contents of acetic acid, propionic acid, and butyric acid in the feces were quantified using the external standard method.

### 2.13. Statistic Analysis

Data are presented as mean ± *SD*. Statistical analyses were conducted using one-way ANOVA followed by Tukey’s post hoc test in SPSS (version 24.0). Groups marked with different letters indicate significant differences (*p* < 0.05). Significance levels are denoted as * *p* < 0.05, ** *p* < 0.01, and *** *p* < 0.001. Graphs were generated using GraphPad Prism (version 8.0.1) and the Majorbio Cloud Platform.

## 3. Results

### 3.1. Comparison of the Effects of DP31 and ADF on Regulating Lipid Metabolism in Obese Mice

**Figure 1:**
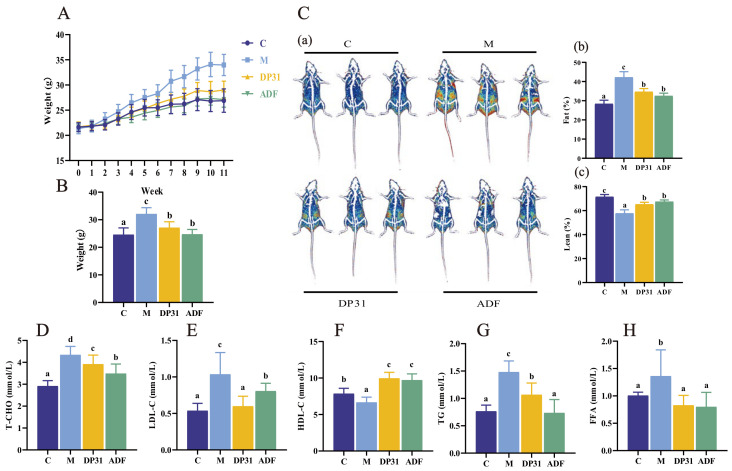
The effect of DP31 and ADF in regulating lipid metabolism in obese mice. (**A**) Body weight (*n* = 10); (**B**) Final body weight (*n* = 8); (**C**) (**a**) Body composition analysis; (**b**) Fat percentage; (**c**) Lean percentage (*n* = 3); (**D**–**G**) Serum concentrations of TG, T-CHO, LDL-C and HDL-C (*n* = 8); (**H**) Serum concentrations of FFA (*n* = 6). Different letters above the bars indicate statistically significant differences between groups (*p* < 0.05), whereas the same letter indicates no significant difference (*p* > 0.05).

Compared with the C group, mice in the M group exhibited significant increases in both body weight and body fat percentage. These metabolic disturbances were markedly ameliorated by both ADF and DP31 interventions. Specifically, both treatments significantly attenuated weight gain and reduced body fat accumulation relative to the M group, with no significant difference in efficacy observed between the two regimens (Figure 1A–C).

HFD feeding significantly increased serum levels of TG, T-CHO, LDL-C, and FFA, while reducing HDL-C levels. Both DP31 and ADF interventions effectively reversed these alterations. Although DP31 was slightly less effective than ADF in reducing TG and T-CHO levels, it demonstrated superior efficacy in lowering LDL-C concentrations. Moreover, DP31 showed effects comparable to those of ADF in normalizing FFA and HDL-C levels (Figure 1D–H). Collectively, these results indicate that DP31 achieves lipid metabolic regulation comparable to that of ADF.

### 3.2. Comparison of the Effects of DP31 and ADF on Regulating Glucose Metabolism in Obese Mice

**Figure 2:**
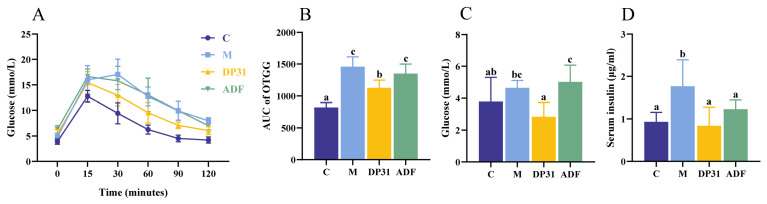
The effect of DP31 and ADF in regulating glucose metabolism in obese mice. (**A**) Blood glucose levels were measured at 0, 15, 30, 60, 90, and 120 min after oral administration of glucose (*n* = 7); (**B**) Area under the oral glucose tolerance test curve; (**C**) Serum concentrations of glucose (*n* = 8); (**D**) Serum concentrations of insulin (*n* = 6). Different letters above the bars indicate statistically significant differences between groups (*p* < 0.05), whereas the same letter indicates no significant difference (*p* > 0.05).

Given the well-established association between lipid and glucose metabolic disorders, we further compared the effects of DP31 and ADF on glucose homeostasis in obese mice (Figure 2A,B). The M group exhibited the most pronounced impairment in glucose tolerance, as reflected by the highest AUC values in the glucose tolerance test (GTT). DP31 intervention significantly improved glucose tolerance compared with the M group, whereas ADF did not produce a statistically significant improvement.

After 11 weeks of intervention, the M group showed the highest fasting blood glucose and insulin levels. Both ADF and DP31 significantly reduced fasting insulin concentrations; however, only DP31 resulted in a significant decrease in fasting blood glucose levels (Figure 2C,D). These results indicate that DP31 not only achieves efficacy comparable to ADF but may also provide superior regulation of glucose metabolism.

### 3.3. Comparison of the Effects of DP31 and ADF on Regulating Liver Lipid Metabolism in Obese Mice

**Figure 3:**
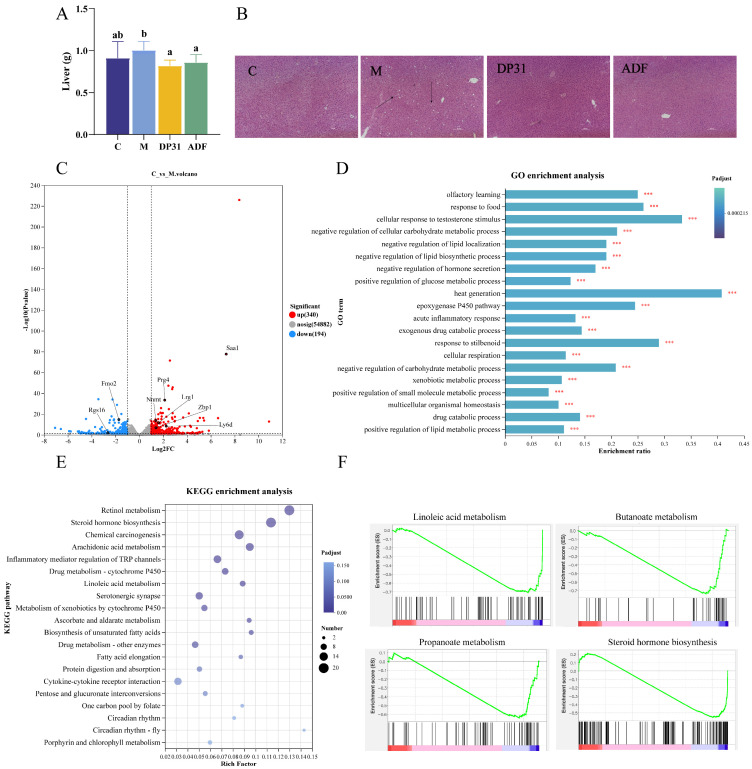
The effect of DP31 and ADF in regulation of liver lipid metabolism in obese mice. (**A**) Weight of Liver (*n* = 8); (**B**) Effects of DP31 replacing ADF on liver tissue morphology in obese mice. Black arrows indicate hepatic lipid vacuoles in the liver tissue (*n* = 3); (**C**) Volcano plot of gene expression differences between the C group and the M group; (**D**) GO enrichment analysis of differentially expressed genes; (**E**) KEGG enrichment analysis of differentially expressed genes; (**F**) GSEA enriched some lipid metabolism and carbohydrates metabolic-related pathways. For transcriptomics analysis (**C**–**F**), *n* = 3. *** refer to the significant difference at *p* < 0.001. Different letters above the bars indicate statistically significant differences between groups (*p* < 0.05), whereas the same letter indicates no significant difference (*p* > 0.05).

The liver plays a central role in lipid metabolism; therefore, hepatic lipid accumulation was evaluated. Consistent with this role, mice in the M group exhibited the greatest liver mass and the highest hepatic TG content, indicating severe hepatic steatosis. Both DP31 and ADF interventions significantly alleviated hepatic lipid deposition (Figure 3A,B). Notably, DP31 achieved a reduction comparable to that observed with ADF, indicating similar efficacy in mitigating liver fat accumulation.

To investigate the molecular mechanisms underlying these phenotypic improvements, hepatic transcriptomic changes were analyzed. Compared with the C group, HFD feeding markedly altered the expression of numerous genes. Specifically, genes associated with lipid accumulation (*Lrg1* [23], *Nnmt* [24], *Ly6d* [25], and *Prg4* [26]) and inflammation (*Saa1* [27], and *Zbp1* [28]) were upregulated, whereas genes involved in fatty acid oxidation (*Fmo2* [29], and *Rgs16* [30]) were downregulated (Figure 3C). Differential expression analysis further indicated that these genes were mainly enriched in biological processes related to the negative regulation of carbohydrate metabolism, lipid localization, lipid biosynthesis, and hormone secretion (Figure 3D). KEGG pathway analysis revealed significant enrichment in retinol metabolism, steroid hormone biosynthesis, chemical carcinogenesis, and arachidonic acid metabolism (Figure 3E). Gene set enrichment analysis (GSEA) further confirmed that HFD feeding resulted in negative enrichment of key metabolic pathways, including linoleic acid metabolism, butanoate metabolism, propanoate metabolism, and steroid hormone biosynthesis (Figure 3F). Collectively, these transcriptomic results demonstrate that HFD feeding profoundly disrupts hepatic metabolic homeostasis.

**Figure 4:**
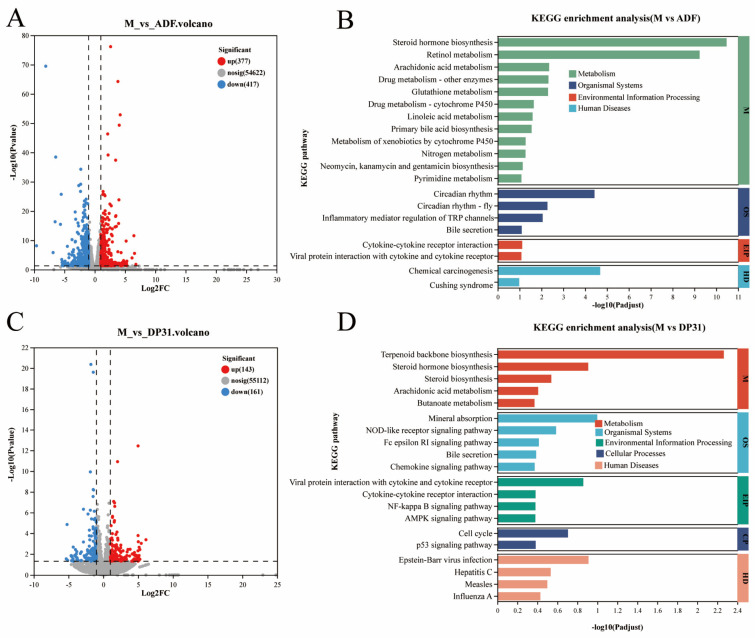
DP31 and ADF jointly affect the effects of high-fat diet feeding on mouse liver gene transcription levels. (**A**) Volcano plot of differentially expressed genes between the ADF group and the M group; (**B**) KEGG enrichment analysis of differentially expressed genes in M vs. ADF; (**C**) Volcano plot of differentially expressed genes between the DP31 group and the M group; (**D**) KEGG enrichment analysis of differentially expressed genes in M vs. DP31. For transcriptomics analysis (**A**–**D**), *n* = 3. *p* < 0.05.

Compared with the M group, ADF intervention resulted in 377 significantly upregulated genes and 417 downregulated genes in the liver (Figure 4A). KEGG enrichment analysis showed that these differentially expressed genes were mainly associated with metabolic processes, biological systems, environmental information processing, and human disease pathways. Among the metabolic pathways, the three most significantly enriched in the ADF group were steroid hormone biosynthesis, retinol metabolism, and arachidonic acid metabolism (Figure 4B). Similarly, DP31 treatment resulted in 143 upregulated and 161 downregulated genes relative to the M group (Figure 4C). The differentially expressed genes in the DP31 group were likewise enriched in pathways related to metabolism, biological systems, environmental information processing, and human diseases. However, the significantly enriched metabolic pathways included terpenoid backbone biosynthesis, steroid hormone biosynthesis, steroid biosynthesis, arachidonic acid metabolism, and butyrate metabolism (Figure 4D). Transcriptome analysis revealed that both ADF and DP31 interventions effectively reversed HFD-induced gene expression disorders. KEGG pathway analysis further indicated that both interventions primarily affected pathways related to steroid metabolism, highlighting a key shared mechanism in their regulation of hepatic gene expression.

**Figure 5:**
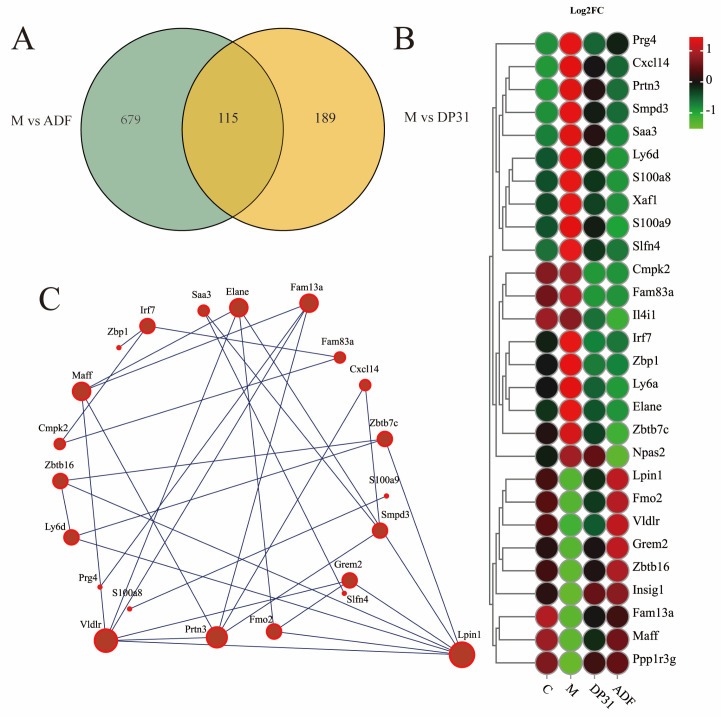
DP31 and ADF jointly affect the effects of high-fat diet feeding on mouse liver gene transcription levels. (**A**) Cross Venn diagram of M vs. DP31 genes and M vs. ADF genes; (**B**) Heat map of genes related to lipid metabolism in the cross genes; (**C**) Correlation between the expression of genes related to lipid metabolism in the cross gene. For transcriptomics analysis (**A**–**C**), *n* = 3. *p* < 0.05.

Both the M vs. ADF and M vs. DP31 comparisons identified 115 co-regulated genes (Figure 5A). Heatmap analysis showed that both ADF and DP31 interventions modulated genes associated with hepatic lipid deposition in HFD-fed mice, including *Fam13a* [31], *Fmo2* [29], *Insig1* [32], *Grem2* [33], *Ppp1r3g* [34], and *Lpin1* [35], as well as inflammation-related genes such as *Cmpk2* [36], *Zbp1* [28], *Il4i1* [37], and *Saa3* [38] (Figure 5B). Correlation analysis revealed that Lpin1 exhibited the strongest association with the expression profiles of the other differentially expressed genes (Figure 5C). *Lpin1* plays a critical role in hepatic de novo lipogenesis and cellular metabolic homeostasis [39,40]. Alternative mRNA splicing generates two major isoforms, Lpin1α and Lpin1β. Lpin1α regulates adipogenic and lipid metabolic gene expression [22]. Previous studies have demonstrated that Lpin1 is a direct substrate of mTORC1, and mTORC1-mediated phosphorylation affects Lpin1α expression while simultaneously activating sterol regulatory element-binding proteins (SREBPs), key transcription factors controlling lipid metabolism [41,42]. Caloric restriction has been reported to regulate Lpin1 activity mainly through modulation of its upstream energy sensor AMPK rather than through direct inhibition of mTORC1 [43,44].

**Figure 6:**
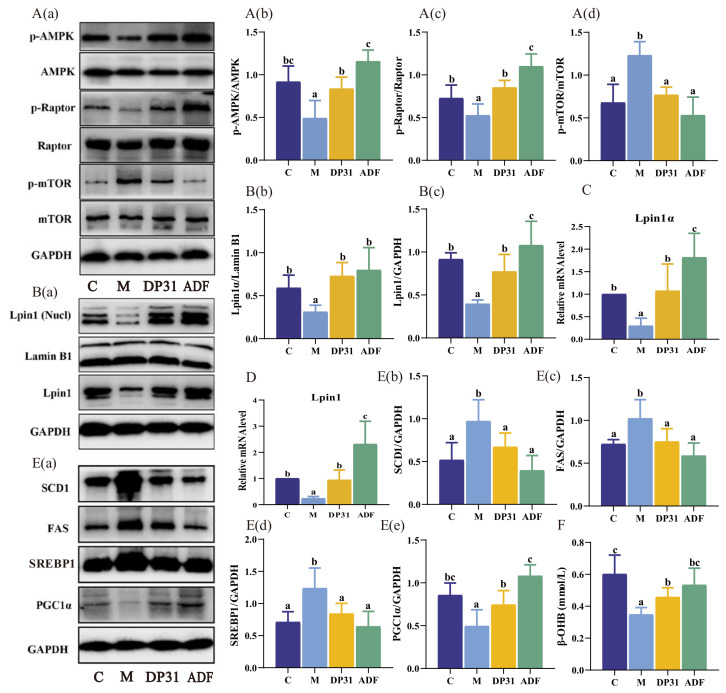
Effects of DP31 and ADF on liver lipoproteins in obese mice. (**A**) Immunoblot and densitometric analysis of p-AMPK, p-mTOR and p-Raptor in mice liver (*n* = 4): (**a**) Representative immunoblot bands of p-AMPK, p-mTOR, p-Raptor; (**b**) Normalized densitometric quantification of p-AMPK expression; (**c**) Normalized densitometric quantification of p-Raptor expression; (**d**) Normalized densitometric quantification of p-mTOR expression; (**B**) Western blot and density analysis of Lpin1 and Lpin1α (*n* = 4): (**a**) Representative immunoblot bands of Lpin1 and Lpin1α; (**b**) Normalized densitometric quantification of Lpin1α expression; (**c**) Normalized densitometric quantification of Lpin1 expression; (**C**) RT-PCR analysis of *Lpin1α* (*n* = 5); (**D**) RT-PCR analysis of *Lpin1*(*n* = 5); (**E**) Western blot and density analysis of SCD1, FAS, SREBP1, and PGC1α (*n* = 4): (**a**) Representative immunoblot bands of SCD1, FAS, SREBP1 and PGC1α; (**b**) Normalized densitometric quantification of SCD1 expression; (**c**) Normalized densitometric quantification of FAS expression; (**d**) Normalized densitometric quantification of SREBP1 expression; (**e**) Normalized densitometric quantification of PGC1α expression; (**F**) Content of β-hydroxybutyrate in serum (*n* = 6). Different letters above the bars indicate statistically significant differences between groups (*p* < 0.05), whereas the same letter indicates no significant difference (*p* > 0.05).

To further investigate the effects of ADF and DP31 on the AMPK–mTORC1 signaling pathway in HFD-induced metabolic dysfunction, we systematically examined the AMPK–mTORC1 signaling axis and its downstream lipid metabolic pathways. HFD feeding suppressed AMPK and Raptor activation while enhancing mTORC1 signaling, and these alterations were significantly reversed by both ADF and DP31 interventions (Figure 6A). In terms of lipogenesis, both interventions increased hepatic Lpin1 expression and increased Lpin1α expression (Figure 6B–D), while suppressing the expression of SREBP1 and its downstream target genes FAS and SCD1 (Figure 6E). Moreover, the HFD-induced reductions in hepatic PGC1α expression and serum β-hydroxybutyrate levels were significantly restored by ADF and DP31 treatments (Figure 6F). These findings suggest that DP31 can substitute for ADF in exhibiting similar effects on specific parameters, coordinately inhibiting hepatic lipogenesis and promoting fatty acid oxidation, while accompanied by changes in the expression of proteins related to the AMPK–mTORC1–Lpin1 signaling axis.

### 3.4. Comparison of DP31 and ADF in Regulating Intestinal Homeostasis in Obese Mice

In addition to the liver, the intestine represents an important target organ for ADF intervention. Furthermore, due to its high molecular weight and high degree of polymerization, DP31 is poorly absorbed in the small intestine. However, it can be degraded by gut microbiota into smaller polyphenolic metabolites, which may act directly on hepatocytes or indirectly exert hepatic effects by modulating the gut microbiota to produce secondary metabolites. Therefore, 16S rRNA gene sequencing was performed to determine whether DP31 exerts effects similar to those of ADF in regulating gut microbiota homeostasis.

**Figure 7:**
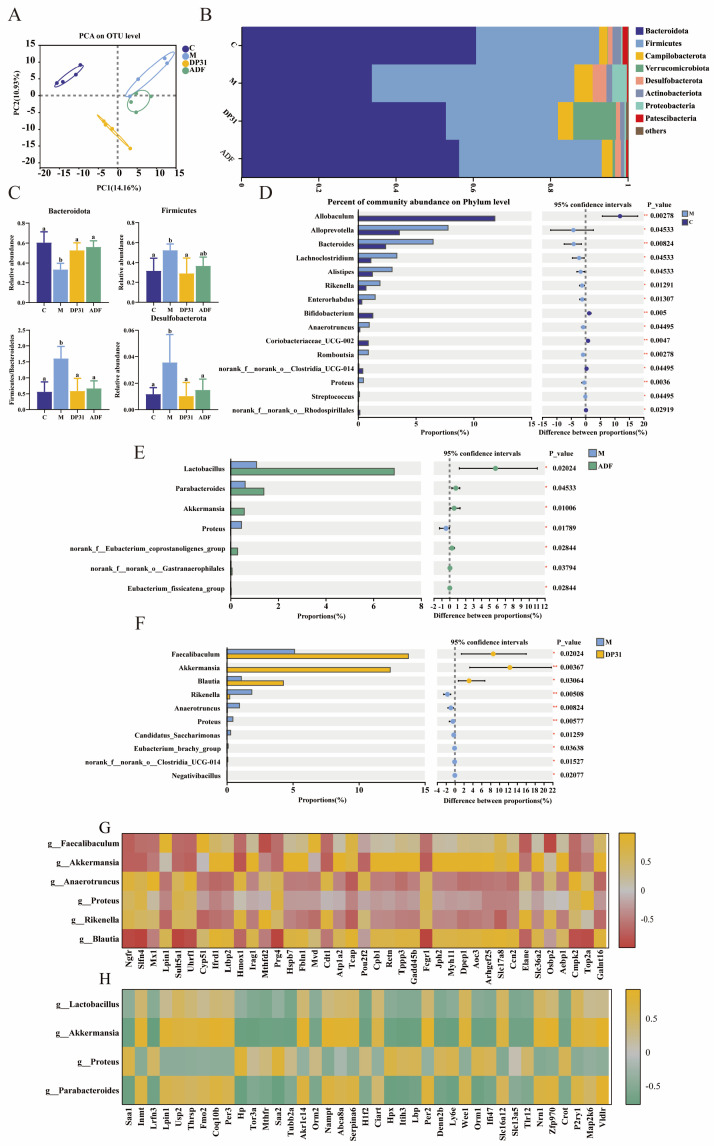
Effects of DP31 and ADF on intestinal homeostasis in obese mice. (**A**) PCA; (**B**) Phylum level composition; (**C**) Relative abundance of Bacteroidetes, Firmicutes, Firmicutes/Bacteroidetes, and Desulfobacterota; (**D**) LDA value distribution histogram between group M and group C; (**E**) LDA value distribution histogram between group M and group ADF; (**F**) LDA value distribution histogram between group M and group DP31; (**G**) Correlation analysis between intestinal microorganisms with high abundance and significant differences before forty in ADF vs. M and genes with significant differences and high content in the liver; (**H**) Correlation analysis between intestinal microorganisms with high abundance and significant differences before forty in DP31 vs. M and genes with significant differences and high content in the liver. The sample size for 16S rRNA sequencing analysis was *n* = 4 per group. Different letters above the bars indicate statistically significant differences between groups (*p* < 0.05), whereas the same letter indicates no significant difference (*p* > 0.05). * and ** refer to the significant difference at *p* < 0.05 and 0.01.

To evaluate overall microbial structural changes, β-diversity was assessed by principal component analysis (PCA). As shown in Figure 7A, the microbial community of the M group was clearly separated from that of the C group, indicating substantial alterations in gut microbiota composition induced by HFD feeding. Notably, both ADF and DP31 interventions shifted the microbial profiles away from the M group, demonstrating effective restoration of the microbial community structure disrupted by HFD.

At the phylum level, Bacteroidetes and Firmicutes accounted for more than 80% of the gut microbiota (Figure 7B), and their relative ratio is considered an important indicator of metabolic status. Compared with the C group, HFD feeding significantly reduced the relative abundance of Bacteroidetes while increasing Firmicutes, resulting in a marked elevation of the Firmicutes/Bacteroidetes (F/B) ratio (Figure 7C). Both ADF and DP31 interventions significantly reversed these alterations by decreasing Firmicutes, increasing Bacteroidetes, and lowering the F/B ratio. In addition, the abundance of Desulfobacterota, which is positively associated with body weight and circulating lipid levels, was significantly lower in both treatment groups compared with the M group.

At the genus level, HFD feeding significantly enriched several bacterial genera compared with the C group, including *Alloprevotella*, *Bacteroides*, *Lachnoclostridium*, *Alistipes*, *Rikenella*, *Enterorhabdus*, *Anaerotruncus*, *Romboutsia,* and *Proteus* (Figure 7D). Relative to the M group, ADF intervention significantly increased the abundance of beneficial genera, including *Lactobacillus*, *Parabacteroides*, *Akkermansia*, *the norank_Eubacterium_coprostanoligenes_group*, *the norank_f_norank_o_Gastranaerophilales*, and the *Eubacterium_fissicatena_group*, while significantly reducing the abundance of *Proteus* (*p* < 0.05; Figure 7E). *Akkermansia*, a mucin-degrading Gram-negative bacterium that colonizes the intestinal mucus layer, produces enzymes that convert mucin into short-chain fatty acids such as acetate and propionate and has been negatively associated with adipose inflammation and hyperlipidemia. In contrast, *Proteus* is widely recognized as a marker of intestinal dysbiosis. These results suggest that ADF is associated with amelioration of HFD-induced intestinal dysfunction, accompanied by enrichment of beneficial bacteria such as *Akkermansia* and suppression of harmful taxa, including *Proteus*. Compared with the M group, DP31 intervention significantly increased the abundance of beneficial genera, particularly *Faecalibaculum* and *Akkermansia*, while reducing several obesity-associated genera, including *Rikenella*, *Anaerotruncus*, *Proteus*, *Candidatus_Saccharimonas*, *Eubacterium_brachy_group*, and *Negativibacillus* (Figure 7F). Notably, DP31 induced a more pronounced increase in *Akkermansia* abundance than ADF, while similarly suppressing *Proteus*.

Increasing evidence highlights the gut-liver axis as a critical link between intestinal microbiota and hepatic metabolic regulation. To explore microbial–host interactions associated with metabolic improvement, correlations were analyzed between differentially abundant microbial taxa and the 40 most significantly altered hepatic genes. In the ADF comparison (Figure 7G), *Lpin1* expression was positively correlated with the abundance of *Lactobacillus*, *Akkermansia*, and *Parabacteroides*, and negatively correlated with *Proteus*. Conversely, the expression of inflammatory genes (*Saa1* and *Saa2*) and lipid-deposition-related genes (*Hp* and *Hpx*) showed negative correlations with beneficial genera and positive correlations with *Proteus*. In the DP31 comparison (Figure 7H), *Lpin1* expression again showed positive correlations with *Faecalibaculum* and *Akkermansia*, and negative correlations with *Anaerotruncus*, *Proteus*, and *Rikenella*. In addition, inflammation-related genes (*Prg4*, *Zbp1*, *Irf7*, and *Ly6d*) were negatively correlated with *Faecalibaculum* and *Akkermansia*, but positively correlated with obesity-associated genera. Collectively, these findings indicate that both ADF and DP31 modulate specific gut microbial populations whose abundances are closely associated with hepatic genes involved in lipid metabolism and inflammatory responses.

### 3.5. Effects of DP31 and ADF on Short-Chain Fatty Acid Content in Feces of Obese Mice

**Figure 8:**
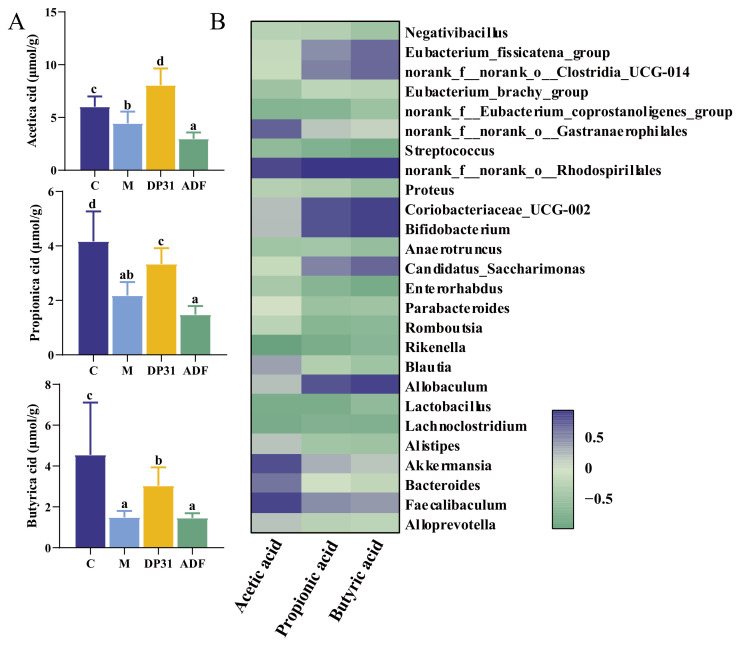
Effects of DP31 and ADF on short-chain fatty acid content in feces of obese mice (*n* = 6). (**A**) Acetic acid, Propionic acid, Butyric acid; (**B**) Analysis of the correlation between short-chain fatty acids and differential intestinal flora. Different letters above the bars indicate statistically significant differences between groups (*p* < 0.05), whereas the same letter indicates no significant difference (*p* > 0.05).

Short-chain fatty acids (SCFAs), primarily acetate, propionate, and butyrate, are key metabolites produced by the gut microbiota. As shown in Figure 8A, fecal concentrations of these SCFAs were significantly reduced in HFD-fed mice compared with the C group. ADF intervention did not restore SCFAs levels and further reduced acetate concentrations. In contrast, DP31 treatment significantly increased fecal SCFAs concentrations. This effect may be attributed to the presence of DP31, which promoted the enrichment of SCFAs-producing genera such as *Faecalibaculum* and *Akkermansia*, whose abundances were positively correlated with total SCFAs levels (Figure 8B). In addition, DP31 may inhibit α-amylase and α-glucosidase activities, thereby increasing the delivery of undigested carbohydrates to the colon and providing additional substrates for microbial fermentation. Taken together, these results suggest that DP31 enhances SCFAs production through coordinated modulation of gut microbial composition and substrate availability.

## 4. Discussion

Obesity is a chronic metabolic disease that poses a major global public health challenge [45]. Various nutritional strategies have been developed to prevent and mitigate obesity [46,47]. Although ADF has been widely recognized as an effective approach for reducing body weight and improving metabolic health, it may also be associated with adverse effects and poor long-term adherence [7,8,9]. Research on alternatives to caloric restriction has largely focused on low-molecular-weight polyphenols, whereas highly polymerized polyphenols remain relatively underexplored. In the present study, transcriptomic analysis and 16S rRNA gene sequencing were employed to evaluate whether highly polymerized persimmon tannin could function as an alternative to ADF in regulating lipid metabolism in obese mice. The aim was to elucidate the underlying mechanisms and to provide a theoretical basis for the development of a practical nutritional strategy for lipid regulation and weight management.

Previous studies have demonstrated that ADF effectively prevents obesity and improves serum lipid profiles [48,49]. Consistent with these findings, the present study showed that ADF significantly attenuated weight gain and body fat accumulation in HFD-fed mice while improving serum lipid parameters. DP31 also exhibited pronounced regulatory effects on body weight and blood lipid levels. Although its effects on TG and T-CHO were slightly weaker than those of ADF, DP31 demonstrated superior efficacy in reducing LDL-C and showed effects comparable to ADF in normalizing FFA and HDL-C levels. These findings indicate that DP31 possesses metabolic regulatory capacity similar to that of ADF in terms of weight control and lipid metabolism.

Glucose metabolism is closely linked to lipid metabolism, and impairment of glucose tolerance has been reported in both rodent and human studies following prolonged ADF intervention. While glucose tolerance may initially improve during short-term ADF intervention, it has been shown to deteriorate with extended treatment [50,51]. Previous studies have reported inconsistent effects of ADF on fasting blood glucose, with some showing significant reductions [52] and others reporting no detectable changes [53]. In the present study, ADF did not significantly improve glucose tolerance or fasting blood glucose levels compared with the M group. These results suggest that although ADF may provide short-term metabolic benefits, prolonged intervention may lead to unfavorable alterations in glucose homeostasis [54]. In contrast, DP31 significantly improved both glucose tolerance and fasting blood glucose levels, indicating that DP31 may offer superior regulation of glucose metabolism compared with ADF.

The liver acts as an essential organ in glucose and lipid metabolism, responsible for maintaining energy homeostasis [55]. The transcriptome results showed that both ADF and DP31 interventions were associated with marked changes in HFD-induced hepatic gene expression. KEGG enrichment analysis identified enrichment of multiple metabolic pathways, including those related to steroid metabolism. Previous studies have demonstrated that caloric restriction can influence the steroid hormone biosynthesis pathway. The liver serves as the primary site for the synthesis and secretion of a variety of steroid hormones, including sex hormones, glucocorticoids, and adrenocortical hormones, and cholesterol is the precursor for the biosynthesis of steroid hormones [56]. The biosynthetic process of steroid hormones involves multiple enzymes, including cytochrome P450s (CYPs) and hydroxysteroid dehydrogenase (HSD) [57]. Among the genes implicated in steroid hormone synthesis, the Hsd17b6 gene, which plays a role in the conversion of testosterone to androstenedione, exhibits lower expression levels in the livers of obese individuals [58]. This finding is consistent with the results of this study. As shown in Supplementary Table S1, the expression of *Hsd17b6* in the liver of mice in group M was down-regulated compared to group C, but was subsequently up-regulated following ADF or DP31 intervention. Furthermore, ADF treatment significantly up-regulated the expression of *Cyp7a1* and *Cyp3a11*. Previous studies have indicated that the up-regulation of *Cyp7a1* and *Cyp3a11* can enhance the biosynthesis of steroid hormones by promoting cholesterol catabolism [59]. Zhang et al. [60] found that ADF can upregulate the expression of *Cyp7a1* in the liver. In addition, the intervention of DP31 in this study also up-regulated the expression of *Cyp7a1* and *Cyp3a11*.

Among the genes co-regulated in the M vs. ADF and M vs. DP31 comparisons, both interventions modulated genes associated with hepatic lipid deposition and inflammation. Fmo2 has been reported to suppress hepatic lipogenesis by inhibiting the transport of SREBP1 from the endoplasmic reticulum to the Golgi apparatus [29]. In our study, both ADF and DP31 significantly upregulated hepatic *Fmo2* expression. Gene co-expression analysis identified *Lpin1* as a central regulatory hub exhibiting the strongest correlations with other metabolic genes. Lpin1 plays a critical role in hepatic de novo lipogenesis and metabolic homeostasis, and its expression has been reported to increase under caloric restriction conditions [39,40]. Similarly, DP31 intervention significantly increased hepatic Lpin1 expression in the present study. Alternative splicing of Lpin1 produces two major isoforms, among which the Lpin1α isoform plays a key role in the regulation of lipid metabolism [22]. Notably, Lpin1 is a direct substrate of mTORC1, and mTORC1-mediated phosphorylation affects Lpin1α expression while promoting SREBP-dependent lipogenic gene expression [41,42]. Caloric restriction primarily regulates this pathway through AMPK activation, which in turn suppresses mTORC1 signaling [43,44]. Consistent with this mechanism, HFD feeding suppressed AMPK activation and enhanced mTORC1 signaling, whereas both ADF and DP31 interventions effectively reversed these alterations. HFD feeding was also associated with reduced total hepatic Lpin1 levels and reduced Lpin1α expression, both of which were restored in association with ADF and DP31 treatments. Furthermore, both interventions significantly suppressed the expression of the key lipogenic transcription factor SREBP1 and its downstream target genes FAS and SCD1, indicating effective inhibition of hepatic de novo lipogenesis. HFD feeding also impaired hepatic fatty acid oxidation, as reflected by reduced PGC1α expression and decreased serum β-hydroxybutyrate levels. Previous studies have shown that increased Lpin1α levels promotes PGC1α expression and enhances mitochondrial fatty acid oxidation. In the present study, restoration of increased Lpin1α levels by both ADF and DP31 was accompanied by increased PGC1α expression and elevated β-hydroxybutyrate levels, suggesting enhanced fatty acid oxidation capacity. Taken together, these findings indicate that DP31 can functionally substitute for ADF in coordinately suppressing hepatic lipogenesis and promoting fatty acid oxidation through modulation of the AMPK-mTORC1-Lpin1 signaling axis, thereby alleviating HFD-induced hepatic lipid metabolic disorders.

Accumulating evidence indicates that gut microbiota dysbiosis contributes to obesity and a range of metabolic disorders [61]. In the present study, both ADF and DP31 interventions effectively ameliorated HFD-induced gut microbiota dysbiosis in mice. ADF treatment notably increased the abundance of beneficial genera, including *Lactobacillus*, *Parabacteroides*, and *Akkermansia*. Previous studies have reported that ADF significantly enhances the abundance of *Lactobacillus* [62]. A genus known to reduce lipid accumulation and oxidative stress, thereby alleviating HFD-induced obesity. *Akkermansia*, frequently recognized as a beneficial bacterium in caloric restriction studies, utilizes mucin as a carbon and nitrogen source and metabolizes it into short-chain fatty acids such as acetate and propionate. Its abundance has been negatively associated with obesity and diabetes. Notably, DP31 produced a more pronounced increase in *Akkermansia* abundance than ADF. Previous studies have demonstrated that enrichment of *Akkermansia* contributes to improved glucose homeostasis in diet-induced obese mice [63]. Therefore, the superior efficacy of DP31 in regulating glucose metabolism observed in this study may be partly attributable to its stronger enrichment of *Akkermansia*. In addition, DP31 intervention significantly increased the abundance of *Faecalibaculum*, a genus whose primary metabolic product is lactate. Lactate-producing gut bacteria have been suggested to exert potential anti-obesity effects [64,65].

Studies have shown that blueberry and blackberry anthocyanins improve metabolic syndrome by regulating gut microbiota and short-chain fatty acid metabolism in HFD-fed C57BL/6J mice [66]. Grape seed polyphenols have also been reported to alleviate obesity by modulating gut microbiota composition and SCFA production [67]. SCFAs, including acetate, propionate, and butyrate, are the principal products of microbial fermentation of dietary fiber [68]. As key microbial metabolites, SCFAs play essential roles in maintaining intestinal homeostasis and regulating metabolic and immune functions, and they also serve as important signaling molecules mediating host–microbiota interactions [69,70]. Studies have shown that dietary supplementation with 5% butyrate prevents and treats obesity and insulin resistance by activating AMPK and inhibiting histone deacetylases, thereby upregulating PGC-1α expression in brown adipose tissue [71]. Acetate has been reported to downregulate ACC and other lipogenesis-related factors. Propionic acid exerts beneficial effects on cholesterol metabolism [72,73]. However, in the present study, ADF intervention did not increase fecal SCFAs concentrations compared with the M group and was associated with a significant reduction in acetate levels. Previous studies have shown that caloric restriction can downregulate enzymes involved in acetate and butyrate synthesis [74], and plasma acetate concentrations have been reported to decrease in overweight or obese individuals following dietary restriction [75]. SCFAs production is influenced by gut microbial composition, substrate availability, and intestinal transit time [76]. The reduction in SCFAs observed during weight loss may reflect decreased efficiency in energy extraction from incompletely digested food. Therefore, the decline in SCFAs levels following ADF intervention is likely attributable to reduced fermentation substrates resulting from lower caloric intake. Several studies have reported that ADF intervention increases SCFAs levels [77]. This discrepancy may be related to the duration of the fasting intervention. In contrast to ADF, DP31 intervention significantly increased fecal SCFAs concentrations in obese mice. This enhancement may be explained by the ability of DP31 to enrich microbial taxa positively associated with SCFAs production, particularly *Akkermansia* [78]. In addition, DP31 may inhibit α-amylase and α-glucosidase activities in the oral cavity and small intestine, thereby increasing the delivery of undigested carbohydrates to the colon and providing additional substrates for microbial fermentation [79]. Notably, no significant changes in the other two major SCFAs were observed under the two different dietary regimes, further supporting that ADF failed to improve SCFAs production. This finding also highlights that DP31 has a distinct and broader impact on gut microbial fermentation by elevating all three major SCFAs. Importantly, animal studies have demonstrated that supplementation with acetate, propionate, or butyrate can reduce blood glucose levels in diabetic mice [80], highlighting the role of SCFAs in glycemic regulation. Therefore, the superior efficacy of DP31 compared with ADF in improving glucose metabolism may be attributed to its stronger enrichment of SCFAs-associated beneficial bacteria and enhanced substrate availability, leading to increased SCFAs production and improved glucose homeostasis.

In this study, C57BL/6J mice were selected as the diet-induced obesity model, a strain that is internationally recognized as the gold standard model. After feeding on a high-fat diet, the mice exhibited metabolic phenotypes closely resembling human obesity, including weight gain, adiposity, dyslipidemia, and hepatic steatosis. This model effectively recapitulates the pathophysiological process of human obesity, and our findings have translational value for the development of human obesity intervention strategies.

Using body surface area normalization, the dose of DP31 (100 mg/kg in mice) is equivalent to approximately 8.1 mg/kg in humans, or about 486 mg/day for a 60 kg adult. Given the low tannin content in fresh persimmon, this dose cannot be achieved through diet alone and would require administration as a dietary supplement or functional food ingredient.

Nevertheless, several limitations should be acknowledged. This study was conducted only in male C57BL/6J mice, limiting generalizability to females, other species, or humans. The 11-week intervention may be insufficient to evaluate long-term safety. Furthermore, the mechanistic findings are correlational; establishing causality will require functional validation, such as pharmacological inhibition, genetic knockout, or fecal microbiota transplantation.

## 5. Conclusions

In conclusion, DP31 exhibits comparable effects to ADF in preventing obesity-related metabolic disorders while demonstrating superior effects on glucose homeostasis. These effects are associated with activation of the AMPK-mTORC1-Lpin1 axis and modulation of gut microbiota. Future studies should focus on clinical validation of DP31 in humans and further mechanistic investigation using pharmacological inhibition or genetic models.

## Data Availability

The original contributions presented in this study are included in the article. Further inquiries can be directed to the corresponding author.

## Supplementary Materials

The following supporting information can be downloaded at: https://www.mdpi.com/article/10.3390/nu18101608/s1, Table S1: Statistics of significantly differentially expressed genes involved in steroid hormone synthesis, linoleic acid metabolism, and retinol metabolism.
