# Supplementary material for: Simulation Effect and Mechanism of High-Polymeric Persimmon Tannin on Simulating Alternate-Day Fasting on Regulating Lipid Metabolism in Obese Mice
Source: Nutrients. 2026 May 18;18(10):1608. doi: 10.3390/nu18101608 (PMC13209242; doi:10.3390/nu18101608)
Supplement: Supplementary file 1 [file nutrients-18-01608-s001.zip › nutrients-4276764-supplementary.pdf]

Supplementary Materials

Table S1. Statistics of significantly differentially expressed genes involved in steroid hormone synthesis, linoleic acid metabolism, and retinol metabolism.

Gene Name	Gene Description	C	M	ADF	DP31
Gpx6	glutathione peroxidase 6	1.777	0.463	1.203	0.760
Cyp2a5	cytochrome P450, family 2, subfamily a, polypeptide 5	161.560	58.840	632.130	99.147
Cyp21a1	cytochrome P450, family 21, subfamily a, polypeptide 1	0.077	0.377	0.167	0.237
Cyp26a1	cytochrome P450, family 26, subfamily a, polypeptide 1	1.400	0.097	1.520	0.343
Cyp2c39	cytochrome P450, family 2, subfamily c, polypeptide 39	1.640	0.747	2.067	1.400
Cyp2c40	cytochrome P450, family 2, subfamily c, polypeptide 40	18.080	3.580	10.117	4.453
Hsd17b6	hydroxysteroid (17-beta) dehydrogenase 6	170.513	104.150	320.883	160.320
Dhrs9	dehydrogenase/reductase (SDR family) member 9	0.123	0.323	0.053	0.213
Cyp7a1	cytochrome P450, family 7, subfamily a, polypeptide 1	14.333	26.197	52.320	27.307
Cyp2b10	cytochrome P450, family 2, subfamily b, polypeptide 10	0.087	0.143	0.703	0.157
Cyp2c38	cytochrome P450, family 2, subfamily c, polypeptide 38	19.323	7.320	19.597	14.843
Akr1c14	aldo-keto reductase family 1, member C14	55.633	34.113	93.367	41.467
Akr1d1	aldo-keto reductase family 1, member D1	44.657	22.180	87.260	32.950
Cyp7b1	cytochrome P450, family 7, subfamily b, polypeptide 1	70.923	192.183	81.293	152.193
Cyp2b9	cytochrome P450, family 2, subfamily b, polypeptide 9	2.547	0.393	43.560	2.663
Akr1c20	aldo-keto reductase family 1, member C20	50.723	31.450	73.947	34.270
Cyp3a11	cytochrome P450, family 3, subfamily a, polypeptide 11	485.170	190.757	593.757	290.263
Cyp2d41-ps	cytochrome P450, family 2, subfamily d, member 41, pseudogene	1.370	3.330	1.347	1.957
Hsd3b3	hydroxy-delta-5-steroid dehydrogenase, 3 beta- and steroid delta-isomerase 3	168.063	135.167	322.307	142.040
Cyp26b1	cytochrome P450, family 26, subfamily b, polypeptide 1	3.720	2.010	4.600	3.493
Hsd3b2	hydroxy-delta-5-steroid dehydrogenase, 3 beta- and steroid delta-isomerase 2	11.190	8.510	24.677	8.570
Cyp4a12a	cytochrome P450, family 4, subfamily a, polypeptide 12a	94.397	201.980	85.920	116.877
Cyp2c54	cytochrome P450, family 2, subfamily c, polypeptide 54	217.520	121.933	266.833	183.970
Cyp2a4	cytochrome P450, family 2, subfamily a, polypeptide 4	0.367	0.013	0.347	0.067
Cyp2c68	cytochrome P450, family 2, subfamily c, polypeptide 68	167.673	55.553	128.580	99.400

Gm16559	predicted gene 16559	0.827	0.393	1.027	0.623
Ugt1a5	UDP glucuronosyltransferase 1 family, polypeptide A5	38.693	11.157	25.533	19.077
Ugt1a10	UDP glycosyltransferase 1 family, polypeptide A10	0.890	0.640	0.093	0.503
Cyp2a22	cytochrome P450, family 2, subfamily a, polypeptide 22	1.937	0.710	4.737	1.153
Cyp2c69	cytochrome P450, family 2, subfamily c, polypeptide 69	3.660	0.393	1.387	1.063
Cyp21a2-ps	cytochrome P450, family 21, subfamily a, polypeptide 2 pseudogene	0.303	1.263	0.617	0.507
Rdh16f1	RDH16 family member 1	0.910	0.117	0.483	0.203
Gm49880	predicted gene, 49880	0.840	0.127	0.987	1.110
